# Avaliação do Nível de Fluxo Coronário com MOTS-C em Pacientes com IAMCSST Submetidos à ICP Primária

**DOI:** 10.36660/abc.20220358

**Published:** 2022-12-20

**Authors:** Tolga Çakmak, Erdoğan Yaşar, Esin Çakmak, Suat Tekin, Yasin Karakuş, Caner Türkoğlu, Furkan Yüksel

**Affiliations:** 1 Departamento de Cardiologia Balikesir Atatürk City Hospital Balikesir Turquia Departamento de Cardiologia – Balikesir Atatürk City Hospital, Balikesir – Turquia; 2 Departamento de Cardiologia Malatya Training and Research Hospital Malatya Turquia Departamento de Cardiologia – Malatya Training and Research Hospital, Malatya – Turquia; 3 Departamento de Saúde Pública Balikesir Provincial Health Department Balikesir Turquia Departamento de Saúde Pública – Balikesir Provincial Health Department, Balikesir – Turquia; 4 Departamento de Fisiologia Inonu University Medical Faculty Malatya Turquia Departamento de Fisiologia – Inonu University Medical Faculty, Malatya – Turquia

**Keywords:** Infarto do miocárdio com supradesnivelamento do segmento ST, Intervenção Coronária Percutânea, Fenômeno de no-reflow, Fases de Leitura Aberta

## Abstract

**Fundamentos:**

Os efeitos protetores da fase de leitura aberta mitocondrial do 12S rRNA-c (MOTS-C) em doenças cardiovasculares foram demonstrados em vários estudos. Entretanto, há pouca documentação da relação entre MOTS-C e fluxo sanguíneo coronariano no infarto do miocárdio com supradesnivelamento do segmento ST (IAMCSST).

**Objetivo:**

Nosso objetivo foi investigar o papel do MOTS-C, que é conhecido por ter propriedades citoprotetoras na patogênese do fenômeno de no-reflow, comparando a taxa de fluxo coronariano e os níveis de MOTS-C em pacientes com IAMCSST submetidos à ICP primária.

**Métodos:**

52 pacientes com IAMCSST e 42 pacientes sem estenose >50% nas artérias coronárias foram incluídos no estudo. O grupo IAMCSST foi dividido em dois grupos de acordo com o grau de fluxo TIMI (do inglês *Thrombolysis In Myocardial Infarction* ) pós-ICP: (i) No-reflow: graus 0, 1 e 2 e (ii) grau 3 (sucesso angiográfico). Um valor de p <0,05 foi considerado significante.

**Resultados:**

Os níveis de MOTS-C foram significativamente menores no grupo IAMCSST em comparação ao grupo controle (91,9 ± 8,9 pg/mL vs. 171,8±12,5 pg/mL, p<0,001). Além disso, a análise da curva *Receiver Operating Characteristics* (ROC) indicou que os níveis séricos de MOTS-C tinham um valor diagnóstico na previsão de no-reflow (Área sob a curva ROC [AUC]: 0,95, IC95%: 0,856-0,993, p < 0,001). Um valor de MOTS-C ≥84,15 pg/mL medido na hospitalização mostrou ter sensibilidade de 95,3% e especificidade de 88,9% na previsão de no-reflow.

**Conclusão:**

MOTS-C é um preditor forte e independente de no-reflow e eventos cardiovasculares adversos maiores (ECAM) intra-hospitalar em pacientes com IAMCSST. Também foi observado que baixos níveis de MOTS-C podem ser um importante marcador prognóstico e podem ter um papel na patogênese do IAMCSST.

## Introdução

O tratamento agudo do infarto do miocárdio com supradesnivelamento do segmento ST (IAMCSST) envolve a abertura da artéria coronária ocluída e a obtenção de reperfusão miocárdica imediata e eficaz. Comparada à fibrinólise, a intervenção coronária percutânea (ICP) primária tem mostrado resultados benéficos quando realizada em até 120 minutos após o diagnóstico em pacientes com IAMCSST, tornando-se a principal estratégia de reperfusão.^1 , 2^ No entanto, a ICP nem sempre oferece resultados benéficos, e uma das complicações frequentemente relatadas da ICP é conhecida como “fenômeno de no-reflow.”^3 , 4^ A patogênese do no-reflow consiste em um processo complexo e dinâmico e é explicada por condições como embolização aterotrombótica distal, lesão isquêmica e lesão de reperfusão.^5^

Os peptídeos derivados da mitocôndria (MDPs, do inglês *mitochondria-derived peptides* ) são novas classes de peptídeos codificados por pequenas fases de leitura aberta (ORFs, do inglês *open reading frames* ) no DNA mitocondrial.^6^ Os MDPs estão amplamente distribuídos em vários tecidos como coração, parede vascular, rim, músculo esquelético e cólon, com esses peptídeos desempenhando papel citoprotetor no organismo através de mecanismos endócrinos e parácrinos, auxiliando na manutenção da função mitocondrial e viabilidade celular, e tendo também efeitos na viabilidade e metabolismo celular, resposta a estressores e inflamação.^7^ Até o momento, três tipos de MDPs foram identificados no corpo humano. Desses, a humanina (HNG) foi o primeiro MDP^8^ descoberto. O segundo peptídeo é conhecido como fase de leitura aberta do rRNA mitocondrial 12S (MOTS-C), que é um peptídeo de 16 aminoácidos codificado por uma ORF pequena dentro do rRNA mitocondrial 12S.^9^ Em períodos posteriores, pequenos peptídeos semelhantes a humanina 1-6 (SHLP1-6) foram descobertos. O MOTS-C estimula a captação de glicose, aumenta a utilização da glicose, oxida ácidos graxos e inibe a respiração oxidativa. Além de seu papel no metabolismo energético, o MOTS-C pode fornecer proteção contra a disfunção endotelial coronariana ao reduzir a liberação de citocinas pró-inflamatórias e moléculas de adesão resultantes da inibição do fator nuclear kappa-B (NF-κB).^10^ Foi hipotetizado que MOTS-C estaria diminuído em pacientes com IAMCSST tratados com intervenção coronária percutânea primária (ICP) e que essa redução poderia ser ainda maior pelo desenvolvimento do fenômeno de no-reflow.

## Materiais e métodos

### População do estudo

**Figura 1:**
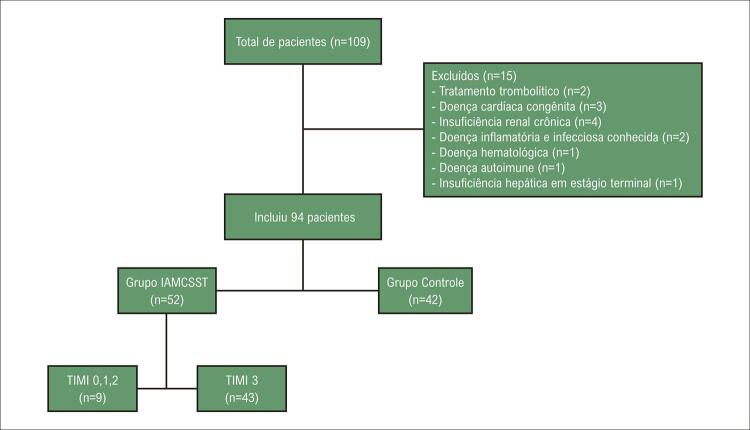
Diagrama de fluxo dos pacientes. IAMCSST: infarto do miocárdio com supradesnivelamento do segmento ST; TIMI: thrombolysis in myocardial infarction.

A metodologia do estudo foi desenhada de acordo com o artigo de Baylan et al. e o número mínimo de pacientes foi determinado de acordo com esse artigo.^11^ O estudo foi realizado em duas etapas, em 94 pacientes que compareceram ao Ambulatório de Cardiologia. O estudo incluiu 52 pacientes consecutivos que apresentaram IAMCSST dentro de seis horas após o início dos sintomas (grupo IAMCSST) e 42 indivíduos controle que não apresentavam estenose grave na angiografia coronária (AC) (grupo controle). O grupo IAMCSST foi composto por pacientes submetidos ao tratamento com ICP primária entre 1º de novembro de 2020 e 15 de fevereiro de 2021. Em contraste o grupo controle foi composto por pacientes que realizaram AC por motivos eletivos entre as mesmas datas, apresentaram estenose inferior a 50% nas artérias coronárias e apresentavam valores normais de troponina na hospitalização. O IAMCSST foi definido como a presença de supradesnivelamento do segmento ST maior que 1mm em pelo menos duas derivações consecutivas no eletrocardiograma (ECG), juntamente com a apresentação de dor torácica típica com duração superior a 30 minutos. O grupo IAMCSST foi dividido em dois grupos de acordo com o grau de fluxo TIMI *(Thrombolysis In Myocardial Infarction* ) pós-ICP;^12^ (i) No-reflow: grau 0, 1 e 2 e (ii) grau 3 (sucesso angiográfico).^13 , 14^ Os critérios de exclusão incluíram história de tratamento trombolítico de IAMCSST nas últimas 24 horas, cardiopatia congênita, insuficiência renal crônica, malignidade conhecida, doença inflamatória conhecida, doença infecciosa, doença hematológica, doença autoimune e insuficiência hepática em estágio terminal ( Figura 1 mostra o diagrama de fluxo dos pacientes).

O estudo foi conduzido de acordo com a Declaração de Helsinque e o protocolo do estudo foi aprovado pelo Comitê de Ética em Pesquisa Clínica da Inonu University Medical School (data de aprovação: 21 de outubro de 2020; nº 2020/160). O consentimento informado foi obtido de cada um dos participantes.

### Angiografia coronária e procedimento de ICP

A AC convencional foi realizada com o sistema Artis Zee montado no piso (Siemens Healthcare, Erlangen, Alemanha) após a hospitalização. Todos os procedimentos de ICP primária foram realizados com cateter-guia 6 e 7F e imagens de AC eletivas foram obtidas com um cateter diagnóstico 6F utilizando a abordagem femoral ou radial padrão. Uma dose de ataque de heparina de 100 UI/kg, ácido acetilsalicílico 100 mg e ticagrelor 180 mg foi administrada aos pacientes submetidos à ICP primária. O implante de stent ou direct stenting foi realizada após a pré-dilatação com balão. A seleção do stent (convencional ou farmacológico) ficou a critério do operador. Em pacientes com fluxo TIMI deficiente, tirofibana intracoronária foi administrada com um bolus inicial de 25 microgramas/kg por um período de três minutos, seguido por uma infusão contínua a uma taxa de 0,15 microgramas/kg/min por 12-24, até 48 horas. Para atingir a dilatação máxima, uma injeção intracoronária de 100 µg de nitroglicerina foi realizada antes de cada angiografia coronariana. O grau TIMI foi avaliado por dois cardiologistas intervencionistas independentes.

### Análise laboratorial e ecocardiografia

Amostras de sangue venoso antecubital foram obtidas de todos os pacientes do grupo IAMCSST durante a internação no departamento de emergência. Amostras de sangue venoso antecubital foram coletadas do grupo controle durante as consultas de seguimento ambulatorial. Os parâmetros lipídicos foram medidos após um período de jejum de 10 horas. A creatinina sérica, glicose sérica, colesterol total sérico, triglicerídeos séricos e lipoproteína de alta densidade sérica-colesterol (HDL-C) foram determinados usando o ensaio Roche (Roche Cobas 6000) com métodos colorimétricos. A lipoproteína de baixa densidade sérica-colesterol (LDL-C) foi calculado indiretamente. Os níveis de MOTS-C foram determinados pelo método ELISA a partir das amostras de soro obtidas após centrifugação das amostras de sangue coletadas diretamente da bainha da artéria femoral ou radial antes da AC em ambos os grupos. Na análise de MOTS-C, kits ELISA específicos para humanos (201-12-8566, Sun Red, China) foram usados para análises de ELISA que foram realizadas no Laboratório de Pesquisa em Fisiologia da Inonu University Medical School. A análise de MOTS-C foi medida em um comprimento de onda de 450 nm (BioTekSynergy HTX, EUA). As análises foram realizadas de acordo com o protocolo do kit e cada amostra foi processada em duplicata. A ecocardiografia transtorácica foi realizada com aparelho de ecocardiografia (VividS60N^®^ GE Medical System, Romênia) com transdutor de 3,5 MHz imediatamente antes do início do procedimento de AC.

### Seguimento e eventos cardíacos adversos maiores

Foram considerados eventos cardíacos adversos maiores (ECAM) a trombose de stent e infarto do miocárdio não-fatal e mortalidade intra-hospitalar durante o período de seguimento intra-hospitalar. A trombose intra-stent foi definida como oclusão angiográfica completa do stent. O infarto do miocárdio não-fatal foi definido como o desenvolvimento de novas alterações no ECG acompanhadas por um novo aumento >20% nos biomarcadores cardíacos medidos após dor torácica recorrente. A mortalidade intra-hospitalar foi definida como morte por infarto do miocárdio, parada cardíaca ou outras causas cardíacas.

### Análise estatística

Os dados foram analisados utilizando SPSS 22.0 para Windows (Armonk, NY: IBM Corp.). A distribuição normal das variáveis contínuas foi avaliada pelo teste de Kolmogorov-Smirnov. As variáveis contínuas com distribuição normal foram expressas como média ± desvio padrão (DP), e as variáveis contínuas com distribuição não-normal como mediana (intervalo interquartil) e comparadas pelo teste *t* de amostras independentes ou teste U de Mann-Whitney, de acordo com a normalidade dos dados. As variáveis categóricas foram expressas em frequências absolutas e relativas e comparadas pelo teste do qui-quadrado. A sensibilidade e especificidade, e o valor de corte de MOTS-C na previsão de fluxo coronariano deficiente após ICP primária em pacientes com IAMCSST foram determinados usando a análise da curva *Receiver Operating Characteristics* (ROC). Foram utilizadas análises de regressão logística univariada e multivariada. Na análise univariada, as variáveis com valor de p <0,25 foram definidas como potenciais fatores de risco para o fenômeno de no-reflow e foram incluídas no modelo completo. Na análise multivariada, os preditores independentes de fluxo TIMI foram analisados através de análise de regressão logística com possíveis fatores identificados em análises anteriores. Um valor de p <0,05 foi considerado significante.

## Resultados

Uma diferença significante foi encontrada entre os grupos IAMCSST e controle em relação à idade e sexo. Destaca-se que a média de idade foi de 59,1±10,9 anos no grupo IAMCSST em contraste com 55,7±8,2 anos no grupo controle (p<0,05). Além disso, embora o gênero masculino tenha sido predominante no grupo IAMCSST, a proporção de mulheres foi significativamente maior no grupo controle (p<0,05). No entanto, não foi encontrada diferença significante entre os dois grupos em relação ao tabagismo, diabetes, hipertensão, história de hiperlipidemia e sinais vitais. Dentre os parâmetros laboratoriais medidos no momento da hospitalização, os níveis de HDL-C foram significativamente maiores no grupo controle (p=0,003), enquanto os níveis de triglicérides (p=0,037), nitrogênio ureico no sangue (BUN), hemoglobina e leucócitos foram significativamente maiores no grupo de pacientes em comparação com o grupo controle. Por outro lado, não foram encontradas diferenças significantes entre os dois grupos em relação a outros parâmetros lipídicos, nível de creatinina e contagem de plaquetas ( Tabela 1 ).

**Tabela 1:** Características demográficas e clínicas

Variável	IAMCSST (n=52)	Controle (n=42)	p
Idade (anos)	59,2 ± 10,9	55,7 ± 8,2	0,04
Sexo (masculino),n(%)	31 (59,6)	16 (38,1)	0,03
Tabagismo, n (%)	24 (46,2)	25 (59,5)	0,27
IMC (kg/m^2^)*	26,3 (24,8-29,0)	28,7 (25,5-31,5)	0,03
Diabetes, n (%)	12 (23,1)	7 (16,7)	0,60
Hipertensão, n (%)	15 (28,8)	9 (21,4)	0,48
Hiperlipidemia, n (%)	10 (19,2)	12 (28,6)	0,41
**Sinais vitais**			
PA Sistólica (mmHg)	133,6 ± 20,6	125,9 ± 16,3	0,06
PA Diastólica (mmHg)	80,8 ± 12,0	81,8 ± 9,2	0,65
FC, batimentos/min	81,6 ± 13,8	78,6 ± 6,6	0,17
**Parâmetros bioquímicos**			
Colesterol total, mg/dL	199,4 ± 46,5	205,0 ± 40,7	0,54
HDL colesterol, mg/dL	39,6 ± 7,9	49,4 ± 8,2	0,00
LDL colesterol, mg/dL	125,1 ± 43,5	127,4 ± 29,4	0,77
Triglicérides séricos, mg/dL*	180,0 (147,5-217,2)	143,5 (95,7-224,2)	0,03
Glicose sérica, mg/dL	154,0 ± 74,5	142,3 ± 73,6	0,44
Nitrogênio da ureia no sangue, mg/dL	35,6 ± 13,0	30,1 ± 11,7	0,03
Creatinina, mg/dL*	0,81 (0,75-0,94)	0,76 (0,68-0,91)	0,15
Hemoglobina (g/dL)	14,5 ± 1,8	13,3 ± 2,0	0,00
Leucócitos, x10^9^/L*	12,2 (9,4-14,8)	6,2 (5,3-8,5)	0,00
Plaquetas, x10^9^/L	258 ± 59	268 ± 67	0,45

*As variáveis contínuas com distribuição normal são expressas como média ± desvio padrão (DP), as variáveis contínuas com distribuição não-normal são expressas como mediana (intervalo interquartil) e as variáveis categóricas são expressas como porcentagem (%). PA: pressão arterial; FC, frequência cardíaca; IMC: índice de massa corporal; HDL: lipoproteína de alta densidade-colesterol; LDL: lipoproteína de baixa densidade-colesterol; IAMCSST: infarto do miocárdio com supradesnivelamento do segmento ST.*

**Figura 2:**
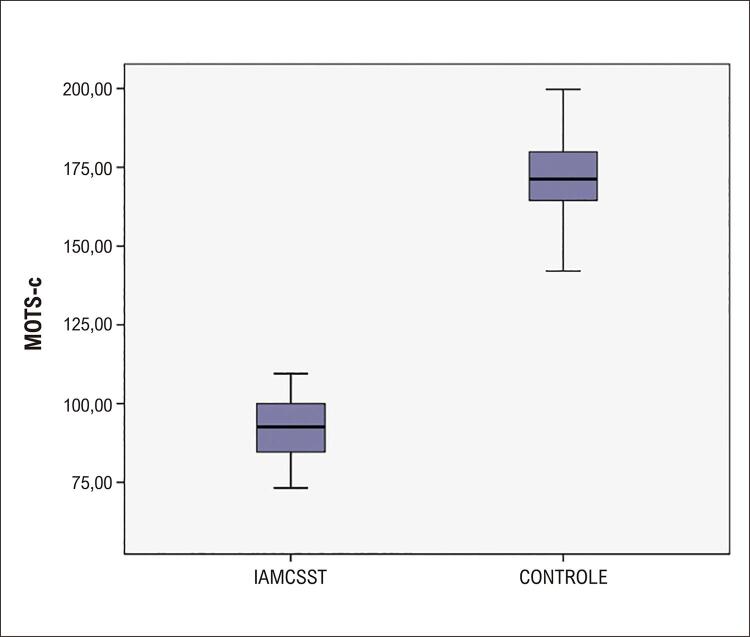
Box-plot dos níveis de MOTS-c nos grupos IAMCSST e controle. MOTS-C: fase de leitura aberta do rRNA mitocondrial 12S; IAMCSST: infarto do miocárdio com supradesnivelamento do segmento ST.

**Figura 3:**
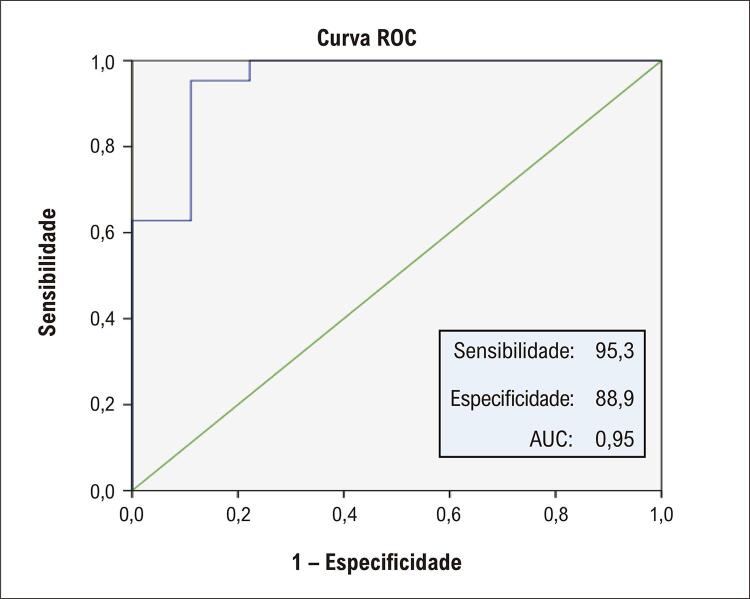
Curva ROC de MOTS-C para previsão do fenômeno de no-reflow angiográfico. MOTS-C: fase de leitura aberta do rRNA mitocondrial 12S; ROC: receiver operating characteristics.

**Figura 4:**
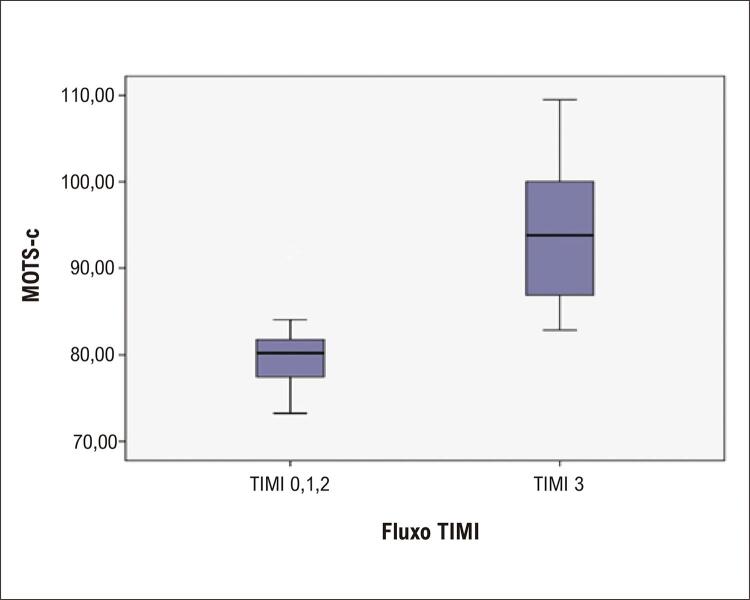
Box-plot do nível de MOTS-C de acordo com o fluxo TIMI (MOTS-C: fase de leitura aberta do rRNA mitocondrial 12S; TIMI: thrombolysis in myocardial infarction.).

Como visto na Figura 2 , os níveis de MOTS-C foram significativamente menores no grupo IAMCSST em comparação com o grupo controle (91,9 ± 8,9 pg/mL vs. 171,8±12,5 pg/mL, p<0,001). Na segunda fase do estudo, o grupo IAMCSST foi dividido em dois grupos de acordo com o grau de fluxo TIMI pós-ICP: (i) No-reflow: grau 0, 1 e 2; e (ii) grau 3 (sucesso angiográfico). Não foram encontradas diferenças significantes entre esses grupos em relação a fatores demográficos, como idade, sexo e histórico de tabagismo, diabetes, hipertensão, hiperlipidemia e histórico de drogas, enquanto o índice de massa corporal (IMC) foi significativamente menor no grupo II e ECAM intra-hospitalar foi significativamente maior no grupo I ( Tabela 2 ). Na análise da curva ROC, o nível sérico de MOTS-C teve um valor diagnóstico na previsão de no-reflow (área sob a curva ROC [AUC]: 0,95, IC95%: 0,856-0,993, p <0,001). Um nível de MOTS-C ≥84,15 pg/mL medido na hospitalização mostrou ter sensibilidade de 95,3% e especificidade de 88,9% na previsão do desenvolvimento de no-reflow ( Figura 3 ). Além disso, o nível de MOTS-C apresentou valor preditivo positivo (VPP) de 97,6% e valor preditivo negativo (VPN) de 80,0%. Como visto na Figura 4 , os níveis de MOTS-C foram significativamente menores nos grupos TIMI 0, 1 e 2, quando comparados ao TIMI 3. Os principais resultados do nosso estudo são mostrados esquematicamente na Figura Central .

**Tabela 2:** Comparação dos grupos TIMI

Variável	TIMI 0, 1, 2 (n=9)	TIMI 3 (n=43)	p
Idade (anos)	54,7 ± 11,2	60,2 ± 10,7	0,17
Sexo (masculino)	4 (44,4)	27 (62,8)	0,45
Tabagismo, n (%)	3 (33,3)	21 (48,8)	0,48
IMC (kg/m^2^)*	29,0 (26,2-31,1)	25,8 (24,2-28,3)	0,03
Diabetes, n (%)	2 (22,2)	10 (23,3)	0,94
Hipertensão, n (%)	2 (22,2)	13 (30,2)	0,71
Hiperlipidemia, n (%)	1 (11,1)	9 (20,9)	0,67
**Medicamentos anteriores, n (%)**			
AAS	1 (11,1)	12 (27,9)	0,42
Beta Bloqueador	0 (00,0)	6 (14,0)	0,35
IECA-BRA	2 (22,2)	12 (27,9)	0,72
Estatina	1 (11,1)	6 (14,0)	0,82
BCC	0 (0,00)	10 (23,3)	0,17
Hidroclorotiazida	1 (11,1)	4 (9,3)	0,86
Furosemida	0 (0,00)	2 (4,7)	0,50
**Sinais vitais**			
PA Sistólica (mmHg)	132,3 ± 22,6	133,9 ± 20,4	0,83
PA Diastólica (mmHg)	78,3 ± 13,2	81,3 ± 11,9	0,50
FC, batimentos/min	81,1 ± 15,2	81,7 ± 13,7	0,90
**Parâmetros bioquímicos**			
Colesterol Total, mg/dL	210,4 ± 26,6	197,1 ± 49,7	0,44
HDL colesterol, mg/dL	41,0 ± 9,4	39,3 ± 7,7	0,64
LDL colesterol, mg/dL	135,9 ± 34,2	122,9 ± 45,2	0,41
Triglicérides séricos, mg/dL*	166,0 (122,0-263,0)	129,0 (89,0-225,0)	0,28
Glicose sérica, mg/dL*	133,0 (101,9-220,5)	127,0 (102,0-178,0)	0,79
Nitrogênio da ureia no sangue, mg/dL	29,8 ± 7,3	36,9 ± 13,6	0,20
Creatinina, mg/dL	0,84 ± 0,11	0,84 ± 0,19	0,83
Hemoglobina (g/dL)	15,1 ± 2,1	14,4 ± 1,8	0,35
Leucócitos, x10^9^/L*	11,3 (9,7-12,9)	12,3 (9,4-15,2)	0,57
Plaquetas, x10^9^/L	238 ± 56	262 ± 59	0,26
**Envolvimento das artérias coronárias**			
Doença uniarterial	3 (33,3)	20 (46,5)	0,71
Doença multiarterial	6 (66,7)	23 (53,5)	0,71
**ICP primária**			
Implante de stent, n (%)	7 (77,8)	43 (100)	0,02
Convencional, n (%)	2 (22,2)	6 (14,0)	0,61
Farmacológico, n (%)	5 (55,6)	37 (86,0)	0,06
Comprimento do stent (mm)	25,8 ± 6,6	24,3 ± 6,7	0,57
Diâmetro do stent (mm)	3,1 ± 0,5	2,9 ± 0,3	0,39
ECAM intra-hospitalar, n (%)	3 (33,3)	1 (2,3)	0,01

*As variáveis contínuas com distribuição normal são expressas como média ± desvio padrão (DP), as variáveis contínuas com distribuição não-normal são expressas como mediana (intervalo interquartil) e as variáveis categóricas são expressas como porcentagem (%). IMC: índice de massa corporal, AAS: ácido acetilsalicílico; IECA: inibidores da enzima conversora da angiotensina; BRA: bloqueadores do receptor de angiotensina; BCC: bloqueador dos canais de cálcio; PA: pressão arterial; HDL: lipoproteína de alta densidade; LDL: lipoproteína de baixa densidade; ECAM: eventos cardíacos adversos maiores; ICP: intervenção coronária percutânea; TIMI: thrombolysis in myocardial infarction.*

**Figura Central:**
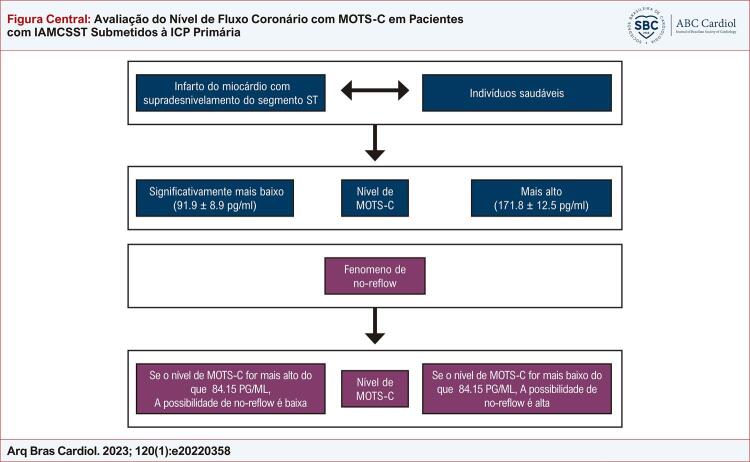
Avaliação do Nível de Fluxo Coronário com MOTS-C em Pacientes com IAMCSST Submetidos à ICP Primária

O grupo IAMCSST foi dividido em dois grupos com base no valor de corte do MOTS-C de 84,15pg/mL e nenhuma diferença significante foi encontrada entre os dois grupos em relação aos fatores de risco demográficos, incluindo idade e sexo e principais fatores de risco, incluindo tabagismo, diabetes, hipertensão e hiperlipidemia. Entretanto, a prevalência de ECAM intra-hospitalar foi significativamente maior no grupo com MOTS-C <84,15 pg/mL ( Tabela 3 ).

**Tabela 3:** Fatores de risco fundamentais de acordo com os níveis de MOTS-C

Variável	MOTS-C< 84,15	MOTS-C ≥ 84,15	p
Idade (anos)	59,4 ± 12,1	59,2 ± 10,7	0,96
Sexo (masculino), n (%)	5 (50,0)	26 (61,9)	0,49
Tabagismo, n (%)	3 (30,0)	21 (50,0)	0,30
IMC (kg/m^2^)*	27,4 (25,3-30,4)	26,2 (24,3-28,5)	0,28
Diabetes, n (%)	2 (20,0)	10 (23,8)	0,79
Hipertensão, n (%)	2 (20,0)	13 (31,0)	0,70
Hiperlipidemia, n (%)	1 (10,0)	9 (21,4)	0,66
Doença uniarterial	4 (40,0)	19 (45,2)	0,76
Doença multiarterial	6 (60,0)	23 (54,8)	0,76
ECAM intra-hospitalar, n (%)	3 (30,0)	1 (2,4)	0,01

*As variáveis contínuas com distribuição normal são expressas como média ± desvio padrão (DP), as variáveis contínuas com distribuição não-normal são expressas como mediana (intervalo interquartil) e as variáveis categóricas são expressas como porcentagem (%). IMC: índice de massa corporal; ECAM: eventos cardíacos adversos maiores; MOTS-C: fase de leitura aberta do rRNA mitocondrial 12S.*

Fatores de risco conhecidos que podem afetar o fluxo coronariano e os níveis de MOTS-C foram analisados por análises de regressão logística univariada e multivariada. Na análise univariada, as variáveis com valor de p < 0,25 foram definidas como potenciais fatores de risco para no-reflow e foram incluídas no modelo completo. Na análise multivariada, o nível de MOTS-C (Odds Ratio [OR]: 2,394, intervalo de confiança de 95% [IC] 1,101-5,205; p = 0,012) foi considerado um fator de risco significante para o fenômeno de no-reflow em pacientes com IAMCSST ( Tabela 4 ).

**Tabela 4:** Análise de regressão logística univariada e multivariada do efeito das variáveis no fluxo TIMI

Varwiável	OR não ajustado	IC95%	p	OR ajustado	IC95%	p
Idade	1,053	0,976-1,137	0,180	1,051	0,926-1,193	0,442
Sexo	2,109	0,493-9,019	0,314			
Tabagismo	1,909	0,422-8,637	0,401			
IMC	0,970	0,789-1,042	0,166	0,754	0,484-1,173	0,210
Diabetes	1,061	0,189-5,943	0,947			
Hipertensão	1,517	0,277-8,310	0,631			
Hiperlipidemia	2,118	0,234-19,20	0,505			
MOTS-C	1,606	1,123-2,296	0,009	2,394	1,101-5,205	0,012

*IMC: índice de massa corporal; IC: intervalo de confiança; MOTS-C: fase de leitura aberta do rRNA mitocondrial 12S; OR: odds ratio.*

## Discussão

Os resultados indicaram que o nível de MOTS-C diminuiu significativamente em pacientes com IAMCSST e essa redução tornou-se ainda mais significante à medida que o nível de fluxo TIMI piorou. Assim, concluiu-se que o baixo nível de MOTS-C pode ser um marcador prognóstico significante de IAMCSST e que MOTS-C pode ter um papel na patogênese do IAMCSST.

Kim et al.,^15^ descobriram que o MOTS-C afeta a conversão de fenótipos secretores associados à senescência (SASPs, do inglês *senescence-associated secretory phenotypes* ) regulando o metabolismo energético mitocondrial e desempenha um papel citoprotetor em doenças relacionadas ao envelhecimento, aliviando assim os sintomas do envelhecimento e melhorando o bem-estar do paciente.^15^ Da mesma forma, André et al.,^16^ mostraram que ao aumentar o fenótipo SASP de MOTS-C, as células senescentes são mais facilmente detectadas e posteriormente eliminadas pelo sistema imunológico, protegendo assim as células normais.^16^ O MOTS-C também é conhecido por desempenhar um papel no metabolismo de aminoácidos, músculos e lipídios.^17^ Qin et al.,^18^ descobriram que pacientes com disfunção endotelial tinham níveis mais baixos de MOTS-C no sangue circulante.^18^ Os MDPs são sintetizados pelo DNA mitocondrial e sua produção é afetada pelo dano mitocondrial. No coração, as mitocôndrias são importantes organelas energizantes e estão envolvidas em vários mecanismos, como estresse oxidativo, autofagia e apoptose.^19^ O MOTS-C pode melhorar o diabetes e outros distúrbios semelhantes, inibindo a resistência à insulina e a obesidade induzida pela dieta.^9^ Tomados em conjunto, todos esses achados indicam que os MDPs desempenham um papel protetor nas doenças cardiovasculares através de diferentes mecanismos. O presente estudo foi inspirado pela ausência de documentação da relação entre IAMCSST e MOTS-C na literatura. Em nosso estudo, os níveis de MOTS-C no grupo IAMCSST foram significativamente menores em comparação com os do grupo controle, o que sugere que um nível baixo de MOTS-C pode desempenhar um papel no desenvolvimento de IAMCSST.

Estudos têm demonstrado a relação entre o fenômeno de no-reflow e a recuperação da função ventricular esquerda, morbidade e mortalidade após infarto agudo do miocárdio.^20^ Alguns outros estudos indicaram que os MDPs desempenham um papel protetor na lesão de isquemia/reperfusão miocárdica. Em um estudo em ratos que avaliou um modelo experimental de isquemia-reperfusão, os animais receberam HNG uma hora antes ou durante a reperfusão e observou-se que a sinalização mediada por óxido nítrico sintase endotelial da proteína quinase AMP-ativada (AMPK) foi ativada e um potencial mecanismo relacionado à regulação de fatores apoptóticos foi eficaz após a administração de HNG. Além disso, também foi observado que as funções ventriculares esquerdas dos camundongos melhoraram e o tamanho do infarto diminuiu à medida que a dose de HNG aumentou.^21^ Após a documentação do efeito da HNG na lesão de isquemia-reperfusão no estudo acima, o presente estudo foi realizado com MOTS-C, que é outro MDP. O presente estudo investigou particularmente a relação entre lesão de isquemia/reperfusão, que tem um papel importante na patogênese do fenômeno de no-reflow e MOTS-C, que tem um efeito protetor conhecido, e mostrou que à medida que o nível de MOTS-C diminui, o fluxo TIMI piora, resultando no fenômeno de no-reflow. Além disso, um nível de MOTS-C ≥84,15 pg/mL medido na hospitalização mostrou ter sensibilidade de 95,3% e especificidade de 88,9% na previsão do desenvolvimento de no-reflow, e níveis elevados de MOTS-C foram considerados um preditor forte e independente do fenômeno de no-reflow e ECAM intra-hospitalar em pacientes com IAMCSST submetidos à ICP primária.

Ming Wei et al.,^22^ mostraram que o tratamento com MOTS-C administrado a ratos levou a uma redução significante na calcificação vascular e pressão arterial, à manutenção da estrutura cardíaca normal, redução da rigidez dos vasos sanguíneos e a um progresso significante na restauração da função cardíaca.^22^ Da mesma forma, o presente estudo também mostrou que a diminuição do nível de MOTS-C pode ter um papel na patogênese do IAMCSST.

Uma atualização recente de Yang et al.,^23^ sugeriu que, como marcadores séricos, os MDPs podem ter um papel diagnóstico nas anormalidades iniciais em doenças cardiovasculares.^23^ No entanto, ainda há um longo caminho a percorrer no que diz respeito ao uso de MDPs para tratamento clínico. Como primeira razão, os mecanismos existentes de MDPs em diferentes doenças cardiovasculares permanecem obscuros e, portanto, são necessários mais estudos para identificar os receptores e as vias de sinalização envolvidas nesses mecanismos. Em segundo lugar, os estudos de MDP em doenças cardiovasculares ainda estão em fase experimental. Além disso, em estudos em animais, não foram observadas reações adversas após a administração de MDPs exógenos.

Por outro lado, os efeitos colaterais ou riscos dos MDPs utilizados para a prevenção ou tratamento de doenças humanas permanecem obscuros; portanto, mais estudos de segurança são necessários.^23^ No presente estudo, uma vez que o papel protetor do MOTS-C no sistema cardiovascular foi demonstrado por vários estudos, apenas os efeitos do MOTS-C no diagnóstico e tratamento do sistema cardiovascular foram investigados. Nossos achados mostraram que o MOTS-C pode ser usado como fator prognóstico no IAMCSST, que é uma das principais causas de mortalidade e morbidade entre as condições cardiovasculares, e também pode ter um papel no tratamento do IAMCSST. Esses achados provavelmente lançarão luz sobre estudos futuros.

### Limitações

O presente estudo foi limitado de várias maneiras. Em primeiro lugar, teve uma pequena população de pacientes e foi realizado em um único centro. Em segundo lugar, os pacientes incluídos no estudo consistiam em pacientes com IAMCSST selecionados aleatoriamente; portanto, nossos achados não podem ser generalizados para toda a população com IAMCSST. Mais e maiores estudos conduzidos com mais biomarcadores são necessários para fundamentar nossos achados na população com IAMCSST e determinar sua generalização para outras populações e etnias.

## Conclusão

Os resultados indicaram que o nível de MOTS-C medido na hospitalização constitui um indicador forte e independente de fluxo sanguíneo coronariano deficiente após a ICP primária. Além disso, um nível de MOTS-C diminuído constitui um preditor forte e independente do fenômeno de no-reflow e ECAM intra-hospitalar.
