# Supplementary material for: Avaliação do Nível de Fluxo Coronário com MOTS-C em Pacientes com IAMCSST Submetidos à ICP Primária
Source: Arq Bras Cardiol. 2022 Dec 20;120(1):e20220358. [Article in Portuguese] doi: 10.36660/abc.20220358 (PMC9833276; doi:10.36660/abc.20220358)
Supplement: Translation

# Translation: Evaluation of Coronary Flow Level with Mots-C in Patients with STEMI Undergoing Primary PCI

**Authors:** Tolga Çakmak, Erdoğan Yaşar, Esin Çakmak, Suat Tekin, Yasin Karakuş, Caner Türkoğlu, Furkan Yüksel

**Affiliations:** 1 Turkey Departamento de Cardiologia – Balikesir Atatürk City Hospital, Balikesir – Turkey; 2 Turkey Departamento de Cardiologia – Malatya Training and Research Hospital, Malatya – Turkey; 3 Turkey Departamento de Saúde Pública – Balikesir Provincial Health Department, Balikesir – Turkey; 4 Turkey Departamento de Fisiologia – Inonu University Medical Faculty, Malatya – Turkey

**Keywords:** ST-elevation myocardial infarction, percutaneous coronary intervention, no-reflow phenomenon, open reading frames

## Abstract

**Background:**

The protective effects of mitochondrial open reading frame of the 12S rRNA-c (MOTS-C) on cardiovascular diseases have been shown in numerous studies. However, there is little documentation of the relationship between MOTS-C and coronary blood flow in ST-segment elevation myocardial infarction (STEMI).

**Objective:**

We aimed to investigate the role of MOTS-C, which is known to have cytoprotective properties in the pathogenesis of the no-reflow phenomenon, by comparing the coronary flow rate and MOTS-C levels in patients with STEMI submitted to primary PCI.

**Methods:**

52 patients with STEMI and 42 patients without stenosis >50% in the coronary arteries were included in the study. The STEMI group was divided into two groups according to post-PCI TIMI (Thrombolysis In Myocardial Infarction) flow grade:(i) No-reflow: grade 0, 1, and 2 and (ii) grade 3(angiographic success). A p value of <0.05 was considered significant.

**Results:**

MOTS-C levels were significantly lower in the STEMI group compared to the control group (91.9 ± 8.9 pg/mL vs. 171.8±12.5 pg/mL, p<0.001). In addition, the Receiver Operating Characteristics (ROC) curve analysis indicated that serum MOTS-C levels had a diagnostic value in predicting no-reflow (Area Under the ROC curve [AUC]:0.95, 95% CI:0.856-0.993, p<0.001). A MOTS-C ≥84.15 pg/mL measured at admission was shown to have 95.3% sensitivity and 88.9% specificity in predicting no-reflow.

**Conclusion:**

MOTS-C is a strong and independent predictor of no-reflow and in-hospital MACE in patients with STEMI. It was also noted that low MOTS-C levels may be an important prognostic marker of and may have a role in the pathogenesis of STEMI.

## Introduction

The acute treatment of ST-segment elevation myocardial infarction (STEMI) involves opening the occluded coronary artery and achieving prompt and effective myocardial reperfusion. Compared to fibrinolysis, primary percutaneous coronary intervention (PCI) has shown beneficial results when performed within 120 minutes of diagnosis in STEMI patients and has thus become the mainstay reperfusion strategy.^1 , 2^However, PCI may not always provide beneficial results, and one of the frequently reported complications of PCI is known as the “no-reflow phenomenon”.^3 , 4^The pathogenesis of no-reflow consists of a complex and dynamic process and is explained by conditions such as distal atherothrombotic embolization, ischemic injury, and reperfusion injury.^5^

Mitochondria-derived peptides (MDPs) are new classes of peptides encoded by small open reading frames (ORFs) in mitochondrial DNA.^6^MDPs are widely distributed in various tissues such as the heart, vascular wall, kidney, skeletal muscle, and colon, with these peptides playing a cytoprotective role in the body through endocrine and paracrine mechanisms, helping to maintain mitochondrial function and cell viability, and also having effects on cell viability and metabolism, response to stressors, and inflammation.^7^To date, three types of MDPs have been identified in the human body. Of these, humanin (HNG) was the first discovered MDP.^8^The second peptide is known as mitochondrial open reading frame of the 12S rRNA-c (MOTS-C), which is a 16-amino-acid peptide encoded by a sORF within the mitochondrial 12S rRNA.^9^In later periods, small humanin-like peptides 1-6 (SHLP1-6) were discovered. MOTS-C stimulates glucose uptake, increases glucose utilization, oxidizes fatty acids, and inhibits oxidative respiration. In addition to its role in energy metabolism, MOTS-C may provide protection against coronary endothelial dysfunction by reducing the release of proinflammatory cytokines and adhesion molecules resulting from nuclear factor kappa B (NF-κB) inhibition.^10^We hypothesized that MOTS-C would decrease in STEMI patients treated with primary percutaneous coronary intervention(PCI) and that this reduction might be further enhanced by the development of no-reflow.

## Materials and Methods

### Study Population

The study methodology was designed according to the article by Baylan et al. and the minimum number of patients was determined according to this article.^11^The study was carried out in two stages in 94 patients who came to the Cardiology Clinic. The study included 52 consecutive patients who presented with STEMI within six hours after symptom onset (STEMI group) and 42 control subjects who had no severe stenosis in the coronary angiography (CAG) (control group). The STEMI group consisted of patients submitted to treatment with primary PCI between November 1, 2020 and February 15, 2021. In contrast, the control group consisted of patients that underwent CAG for elective reasons between the same dates, had less than 50% stenosis in the coronary arteries, and had normal troponin values on admission. STEMI was defined as the presence of ST-elevation greater than 1 mm in at least two consecutive leads on the electrocardiogram (ECG) along with the presentation of a typical chest pain lasting greater than 30 minutes. The STEMI group was divided into two groups according to post-PCI TIMI (Thrombolysis In Myocardial Infarction) flow grade:^12^(i) No-reflow: grade 0, 1, and 2 and (ii) grade 3 (angiographic success).^13 , 14^The exclusion criteria included a history of thrombolytic treatment of STEMI within the last 24 hours, congenital heart disease, chronic renal failure, known malignancy, known inflammatory disease, infectious disease, hematological disease, autoimmune disease, and end-stage liver failure ( Figure 1 shows the patient flow diagram).

**Figure 1:** Patient flow diagram. STEMI: ST-segment elevation myocardial infarction; TIMI: thrombolysis in myocardial infarction.

The study was conducted in accordance with the Declaration of Helsinki and the study protocol was approved by Inonu University Medical School Clinical Research Ethics Committee (Approval date: October 21, 2020; No. 2020/160). Informed consent was obtained from each participant.

### Coronary angiography and PCI procedure

Conventional CAG was performed with a Siemens Artis zee floor-mounted system (Siemens Healthcare, Erlangen, Germany) following admission. All the primary PCI procedures were performed with a 6- and 7-F guiding catheter and elective CAG images were obtained with a 6-F diagnostic catheter using the standard femoral or radial approach. A loading dose of heparin of 100 IU/kg, acetylsalicylic acid 100 mg, and ticagrelor 180 mg was administered to patients submitted to primary PCI. Stenting or direct stenting was performed following balloon pre-dilatation. Stent selection (bare metal or drug-eluting) was left to the discretion of the operator. In patients with poor TIMI flow, intracoronary tirofiban was administered with an initial bolus of 25 microgram/kg given over a 3-minute period, followed by a continuous infusion at a rate of 0.15 microgram/kg/min for 12-24, up to 48 hours. To achieve maximum dilatation, an intracoronary injection of 100 µg nitroglycerin was performed before each coronary angiogram. TIMI grade was assessed by two independent interventional cardiologists.

### Laboratory analysis and echocardiography

Antecubital venous blood samples were obtained from all patients in the STEMI group during the admission to the emergency department. Antecubital venous blood samples were taken from the control group during the outpatient follow-up visits. Lipid parameters were measured after a 10-hour fasting period. Serum creatinine, serum glucose, serum total cholesterol, serum triglycerides, and serum high-density lipoprotein cholesterol (HDL-C) were determined using the Roche assay (Roche Cobas 6000) with colorimetric methods. Serum low-density lipoprotein cholesterol (LDL-C) was calculated indirectly. MOTS-C levels were determined using the ELISA method from the serum samples that were obtained after centrifuging the blood samples collected directly from the femoral or radial artery sheath prior to CAG in both groups. In the MOTS-C analysis, human-specific ELISA kits (201-12-8566, Sun Red, China) were used for ELISA analyses that were performed at Inonu University Medical School Physiology Research Laboratory. MOTS-C analysis was measured at a wavelength of 450 nm (BioTekSynergy HTX, USA). The analyses were performed in accordance with the kit protocol and each sample was run in duplicate. Transthoracic echocardiography was performed using an echocardiography device (VividS60N® GE Medical System, Romania) with a 3.5-MHz transducer immediately before starting the CAG procedure.

### Follow-up and major adverse cardiac events

Major adverse cardiac events (MACE) were considered as stent thrombosis and non-fatal myocardial infarction, and in-hospital mortality during the in-hospital follow-up period. Intra-stent thrombosis was defined as complete angiographic occlusion of the stent. Non-fatal myocardial infarction was defined as the development of new ECG changes accompanied by a new rise >20% in measured cardiac biomarkers after recurrent chest pain. In-hospital mortality was defined as death from myocardial infarction, cardiac arrest, or other cardiac causes.

### Statistical analysis

Data were analyzed using SPSS 22.0for Windows (Armonk, NY: IBM Corp.). The normal distribution of continuous variables was assessed using the Kolmogorov-Smirnov test. Normally distributed continuous variables were expressed as mean ± standard deviation (SD), and non-normally distributed continuous variables as median (interquartile range) and compared using Independent samples t-test or Mann-Whitney U-test, according to data normality. Categorical variables were expressed as absolute and relative frequencies and were compared using the Chi-square test. The sensitivity and specificity, and the cut-off value of MOTS-C in predicting poor coronary flow after primary PCI in patients with STEMI were determined using the Receiver Operating Characteristics (ROC) curve analysis. Univariate and multivariate logistic regression analyses were used. In the univariate analysis, variables with a p value <0.25 were defined as potential risk factors for no-reflow and were included in the full model. In the multivariate analysis, independent predictors of TIMI flow were analyzed using logistic regression analysis with possible factors identified in previous analyses. A p value <0.05 was considered significant.

## Results

A significant difference was found between the STEMI and control groups with regard to age and gender. Of note, the mean age was 59.1±10.9 years in the STEMI group as opposed to 55.7±8.2 years in the control group (p<0.05). Moreover, although male gender was dominant in the STEMI group, the proportion of females was significantly higher in the control group (p<0.05). However, no significant difference was found between the two groups with regard to smoking status, diabetes, hypertension, history of hyperlipidemia, and vital signs. Among the laboratory parameters measured at the time of admission, HDL-C levels were significantly higher in the control group (p=0.003), whereas triglycerides (p=0.037), blood urea nitrogen (BUN), hemoglobin, and leukocyte levels were significantly higher in the patient group compared to the control group. On the other hand, no significant differences were found between the two groups regarding other lipid parameters, creatinine level, and platelet count ( Table 1 ).

**Table 1:** Demographic and clinical characteristics

Variable	STEMI (n=52)	Control (n=42)	p
Age (years)	59.2 ± 10.9	55.7 ± 8.2	0.04
Gender (male),n(%)	31 (59.6)	16 (38.1)	0.03
Smoking status, n (%)	24 (46.2)	25 (59.5)	0.27
BMI (kg/m2)*	26.3 (24.8-29.0)	28.7 (25.5-31.5)	0.03
Diabetes, n (%)	12 (23.1)	7 (16.7)	0.60
Hypertension, n (%)	15 (28.8)	9 (21.4)	0.48
Hyperlipidemia, n (%)	10 (19.2)	12 (28.6)	0.41
**Vital signs**			
Systolic BP (mmHg)	133.6 ± 20.6	125.9 ± 16.3	0.06
Diastolic BP (mmHg)	80.8 ± 12.0	81.8 ± 9.2	0.65
Heart rate, beats/min	81.6 ± 13.8	78.6 ± 6.6	0.17
**Biochemical parameters**			
Total cholesterol, mg/dL	199.4 ± 46.5	205.0 ± 40.7	0.54
HDL cholesterol, mg/dL	39.6 ± 7.9	49.4 ± 8.2	0.00
LDL cholesterol, mg/dL	125.1 ± 43.5	127.4 ± 29.4	0.77
Serum triglyceride, mg/dL*	180.0 (147.5-217.2)	143.5 (95.7-224.2)	0.03
Serum glucose, mg/dL	154.0 ± 74.5	142.3 ± 73.6	0.44
Blood urea nitrogen, mg/dL	35.6 ± 13.0	30.1 ± 11.7	0.03
Creatinine, mg/dL*	0.81 (0.75-0.94)	0.76 (0.68-0.91)	0.15
Hemoglobin (g/dL)	14.5 ± 1.8	13.3 ± 2.0	0.00
Leukocytes, x109/L*	12.2 (9.4-14.8)	6.2 (5.3-8.5)	0.00
Platelets, x109/L	258 ± 59	268 ± 67	0.45

As seen in Figure 2 , MOTS-C levels were significantly lower in the STEMI group compared to the control group (91.9 ± 8.9 pg/mL *vs.* 171.8±12.5 pg/mL, *p* <0.001). In the second phase of the study, the STEMI group was divided into two groups according to post-PCI TIMI flow grade: (i) No-reflow: grade 0, 1, and 2 and (ii) grade 3 (angiographic success). No significant differences were found between these groups regarding demographic factors such as age, gender, and history of smoking, diabetes, hypertension, hyperlipidemia, and drug history, whereas body mass index (BMI) was significantly lower in group II and in-hospital MACE was significantly higher in group I ( Table 2 ). In the ROC analysis, serum MOTS-C level had a diagnostic value in predicting no-reflow (Area Under the ROC curve [AUC]: 0.95, 95% CI: 0.856-0.993, p<0.001). A MOTS-C level of ≥84.15 pg/mL measured at admission was found to have 95.3% sensitivity and 88.9% specificity in predicting the development of no-reflow ( Figure 3 ). Additionally, the MOTS-C level had a positive predictive value (PPV) of 97.6% and a negative predictive value (NPV) of 80.0%. As seen in Figure 4 , MOTS-C levels were significantly lower in the TIMI 0, 1, and 2 groups, when compared to TIMI 3. The main results of our study are shown schematically in Central illustration .

**Figure 2:** Patient flow diagram. STEMI: ST-segment elevation myocardial infarction; TIMI: thrombolysis in myocardial infarction.

**Table 2:** Comparison of TIMI groups

Variable	TIMI 0, 1, 2 (n=9)	TIMI 3 (n=43)	p
Age (years)	54.7 ± 11.2	60.2 ± 10.7	0.17
Gender (male)	4 (44.4)	27 (62.8)	0.45
Smoking status, n (%)	3 (33.3)	21 (48.8)	0.48
BMI (kg/m2)*	29.0 (26.2-31.1)	25.8 (24.2-28.3)	0.03
Diabetes, n (%)	2 (22.2)	10 (23.3)	0.94
Hypertension, n (%)	2 (22.2)	13 (30.2)	0.71
Hyperlipidemia, n (%)	1 (11.1)	9 (20.9)	0.67
**Previous medications, n (%)**			
ASA	1 (11.1)	12 (27.9)	0.42
Beta Blocker	0 (00.0)	6 (14.0)	0.35
ACEI-ARB	2 (22.2)	12 (27.9)	0.72
Statin	1 (11.1)	6 (14.0)	0.82
Ca CB	0 (0.00)	10 (23.3)	0.17
Hydrochlorothiazide	1 (11.1)	4 (9.3)	0.86
Furosemide	0 (0.00)	2 (4.7)	0.50
**Vital signs**			
Systolic BP (mmHg)	132.3 ± 22.6	133.9 ± 20.4	0.83
Diastolic BP (mmHg)	78.3 ± 13.2	81.3 ± 11.9	0.50
Heart rate, beats/min	81.1 ± 15.2	81.7 ± 13.7	0.90
**Biochemical parameters**			
Total cholesterol, mg/dL	210.4 ± 26.6	197.1 ± 49.7	0.44
HDL cholesterol, mg/dL	41.0 ± 9.4	39.3 ± 7.7	0.64
LDL cholesterol, mg/dL	135.9 ± 34.2	122.9 ± 45.2	0.41
Serum triglycerides, mg/dL*	166.0 (122.0-263.0)	129.0 (89.0-225.0)	0.28
Serum glucose, mg/dL*	133.0 (101.9-220.5)	127.0 (102.0-178.0)	0.79
Blood urea nitrogen, mg/dL	29.8 ± 7.3	36.9 ± 13.6	0.20
Creatinine, mg/dL	0.84 ± 0.11	0.84 ± 0.19	0.83
Hemoglobin (g/dL)	15.1 ± 2.1	14.4 ± 1.8	0.35
Leukocytes, x109/L*	11.3 (9.7-12.9)	12.3 (9.4-15.2)	0.57
Platelets, x109/L	238 ± 56	262 ± 59	0.26
**Coronary artery involvement**			
Single-vessel disease	3 (33.3)	20 (46.5)	0.71
Multivessel disease	6 (66.7)	23 (53.5)	0.71
**Primary PCI**			
Stent implantation, n (%)	7 (77.8)	43 (100)	0.02
BMS, n (%)	2 (22.2)	6 (14.0)	0.61
DES, n (%)	5 (55.6)	37 (86.0)	0.06
Stent length (mm)	25.8 ± 6.6	24.3 ± 6.7	0.57
Stent diameter (mm)	3.1 ± 0.5	2.9 ± 0.3	0.39
In-hospital MACE, n (%)	3 (33.3)	1 (2.3)	0.01

*Normally distributed continuous variables are expressed as mean ± standard deviation (SD), non-normally distributed continuous variables are expressed as median (interquartile range) and categorical variables are expressed as percent (%). BMI: body mass index, ASA: acetylsalicylic acid; ACEI: angiotensin-converting-enzyme inhibitors; ARB: angiotensin receptor blockers; BMS: bare metal stent; Ca CB: calcium channel blocker; BP: blood pressure; DES: drug-eluting stent; HDL: high-density lipoprotein; LDL: low-density lipoprotein; MACE: major adverse cardiac events; PCI: percutaneous coronary intervention; TIMI: thrombolysis in myocardial infarction.*

**Figure 3:** ROC curve of MOTS-C for predicting angiographic no-reflow. MOTS-C: mitochondrial open reading frame of the 12S rRNA-c; ROC: receiver-operating characteristic.

**Figure 4:** Box-plot of MOTS-C level according to TIMI flow MOTS-C: mitochondrial open reading frame of the 12S rRNA-c; TIMI: thrombolysis in myocardial infarction.

**Central Illustration:** Evaluation of Coronary Flow Level with MOTS-C in Patients with STEMI Undergoing Primary PCI

The STEMI group was divided into two groups based on the cut-off MOTS-C value of 84.15pg/mL and no significant difference was found between the two groups regarding demographic risk factors, including age and gender and major risk factors including smoking status, diabetes, hypertension, and hyperlipidemia. However, the prevalence of in-hospital MACE was significantly higher in the group with MOTS-C<84.15 pg/mL ( Table 3 ).

**Table 3:** Fundamental risk factors according to MOTS-C levels

Variable	MOTS-C< 84.15	MOTS-C ≥ 84.15	p
Age (years)	59.4 ± 12.1	59.2 ± 10.7	0.96
Gender (male), n (%)	5 (50.0)	26 (61.9)	0.49
Smoking status, n (%)	3 (30.0)	21 (50.0)	0.30
BMI (kg/m^2^)*	27.4 (25.3-30.4)	26.2 (24.3-28.5)	0.28
Diabetes, n (%)	2 (20.0)	10 (23.8)	0.79
Hypertension, n (%)	2 (20.0)	13 (31.0)	0.70
Hyperlipidemia, n (%)	1 (10.0)	9 (21.4)	0.66
Single-vessel disease	4 (40.0)	19 (45.2)	0.76
Multivessel disease	6 (60.0)	23 (54.8)	0.76
In-hospital MACE, n (%)	3 (30.0)	1 (2.4)	0.01

*Normally distributed continuous variables are expressed as mean ± standard deviation (SD), non-normally distributed continuous variables are expressed as median (interquartile range) and categorical variables are expressed as percent (%). BMI: body mass index; MACE: major adverse cardiac events; MOTS-C: mitochondrial open reading frame of the 12S rRNA-c.*

Known risk factors that may affect coronary flow and MOTS-C levels were analyzed by univariate and multivariate logistic regression analyses. In the univariate analysis, variables with a p value <0.25 were defined as potential risk factors for no-reflow and were included in the full model. In the multivariate analysis, MOTS-C level (Odds Ratio [OR]: 2.394, 95% confidence interval [CI] 1.101-5.205; p=0.012) was found to be a significant risk factor for no-reflow in STEMI patients ( Table 4 ).

**Table 4:** Univariate and multivariate logistic regression analysis of the effect of variables on TIMI flow

Variable	Unadjusted OR	95% Cl	p	Adjusted OR	95% Cl	p
Age	1.053	0.976-1.137	0.180	1.051	0.926-1.193	0.442
Gender	2.109	0.493-9.019	0.314			
Smoking status	1.909	0.422-8.637	0.401			
BMI	0.970	0.789-1.042	0.166	0.754	0.484-1.173	0.210
Diabetes	1.061	0.189-5.943	0.947			
Hypertension	1.517	0.277-8.310	0.631			
Hyperlipidemia	2.118	0.234-19.20	0.505			
MOTS-C	1.606	1.123-2.296	0.009	2.394	1.101-5.205	0.012

*BMI: body mass index; CI: confidence interval; MOTS-C: mitochondrial open reading frame of the 12S rRNA-c; OR: Odds ratio.*

## Discussion

The results indicated that the MOTS-C level decreased significantly in STEMI patients and this reduction became even more significant as the TIMI flow level worsened. Accordingly, it was concluded that low MOTS-C level may be a significant prognostic marker of STEMI and that MOTS-C may have a role in the pathogenesis of STEMI.

Kim et al.^15^found that MOTS-C affects the conversion of senescence-associated secretory phenotypes (SASPs) by regulating mitochondrial energy metabolism and plays a cytoprotective role in aging-related diseases, thereby relieving aging symptoms and improving patient well-being.^15^Similarly, Andrew et al.^16^showed that by increasing the SASP phenotype of MOTS-C, senescent cells are more easily detected and subsequently cleared by the immune system, thereby protecting normal cells.^16^MOTS-C is also known to play a role in amino acid, muscle, and lipid metabolism.^17^Qin et al.^18^found that patients with endothelial dysfunction had lower levels of MOTS-C in circulating blood.^18^MDPs are synthesized by mitochondrial DNA and their production is affected by mitochondrial damage. In the heart, mitochondria are important energizing organelles and are involved in various mechanisms, such as oxidative stress, autophagy, and apoptosis.^19^MOTS-C can ameliorate diabetes and other similar disorders by inhibiting insulin resistance and diet-induced obesity.^9^Taken together, all these findings indicate that MDPs play a protective role in cardiovascular diseases through different mechanisms. The present study was inspired by the absence of the documentation of the relationship between STEMI and MOTS-C in the literature. In our study, the MOTS-C levels in the STEMI group were significantly lower compared to those of control group, which suggests that low MOTS-C may play a role in the development of STEMI.

Studies have shown a relationship between the no-reflow phenomenon and the recovery of left ventricular function, morbidity and mortality after acute myocardial infarction.^20^Some other studies indicated that MDPs play a protective role in myocardial ischemia/reperfusion injury. In a rat study that evaluated an experimental ischemia-reperfusion model, the animals were administered HNG one hour before or during reperfusion and it was observed that AMP-activated protein kinase (AMPK)-endothelial nitric oxide synthase-mediated signaling was activated and a potential mechanism related to the regulation of apoptotic factors was effective after the administration of HNG. Additionally, it was also observed that the left ventricular functions of the mice improved and the infarct size decreased as the HNG dose increased.^21^Following the documentation of the effect of HNG on ischemia-reperfusion injury in the above study, the present study was conducted with MOTS-C, which is another MDP. The present study particularly investigated the relationship between ischemia/reperfusion injury, which has an important role in the pathogenesis of the no-reflow phenomenon and MOTS-C, which has a known protective effect, and it was shown that as the MOTS-C level decreases, the TIMI flow worsens, ultimately resulting in the no-reflow phenomenon. Additionally, a MOTS-C level ≥84.15 pg/mL measured at admission was found to have 95.3% sensitivity and 88.9% specificity in predicting the development of no-reflow, and elevated MOTS-C levels were found to be a strong and independent predictor of the no-reflow phenomenon and in-hospital MACE in STEMI patients undergoing primary PCI.

Ming Wei et al.^22^showed that MOTS-C treatment administered to rats led to a significant reduction in vascular calcification and blood pressure, maintenance of normal cardiac structure, reduced blood vessel stiffness, and a significant progress in cardiac function restoration.^22^Similarly, the present study also showed that the decrease in MOTS-C level may have a role in the pathogenesis of STEMI.

A recent update by Yang et al.^23^suggested that as serum markers, MDPs may have a diagnostic role in early abnormalities in cardiovascular diseases.^23^Nevertheless, there is still a long way to go regarding the use of MDPs for clinical treatment. As the first reason, the existing mechanisms of MDPs in different cardiovascular diseases remain unclear and, thus, further studies are needed to identify the receptors and signaling pathways involved in these mechanisms. Second, MDP studies in cardiovascular diseases are still in the experimental stage. Additionally, in animal studies, no adverse reactions were observed following the administration of exogenous MDPs.

On the other hand, the side effects or risks of MDPs used for the prevention or treatment of human diseases remain unclear; therefore, further safety studies are needed.^23^In the present study, since the protective role of MOTS-C on the cardiovascular system had been demonstrated by numerous studies, only the effects of MOTS-C on the diagnosis and treatment of cardiovascular system was investigated. Our findings showed that MOTS-C could be used as a prognostic factor in STEMI, which is a leading cause of mortality and morbidity among cardiovascular conditions, and it may also have a role in STEMI treatment. These findings are likely to shed light on future studies.

### Limitations

The present study was limited in several ways. First and foremost, it had a small patient population and was conducted in a single center. Secondly, the patients included in the study consisted of randomly selected STEMI patients; therefore, our findings cannot be generalized to the entire STEMI population. Further larger studies conducted with more biomarkers are needed to substantiate our findings in the STEMI population and to determine their generalizability to other populations and ethnicities.

## Conclusion

The results indicated that the MOTS-C level measured at admission constitutes a strong and independent indicator of poor coronary blood flow following primary PCI. Additionally, a decreased MOTS-C level constitutes a strong and independent predictor of no-reflow and in-hospital MACE.
